# The complete genome sequence of the rumen methanogen *Methanosarcina barkeri* CM1

**DOI:** 10.1186/s40793-015-0038-5

**Published:** 2015-08-19

**Authors:** Suzanne C. Lambie, William J. Kelly, Sinead C. Leahy, Dong Li, Kerri Reilly, Tim A. McAllister, Edith R. Valle, Graeme T. Attwood, Eric Altermann

**Affiliations:** Rumen Microbiology, Animal Nutrition and Health, AgResearch Limited, Tennent Drive, Private Bag 11008, Palmerston North, 4442 New Zealand; New Zealand Agricultural Greenhouse Gas Research Centre, Grasslands Research Centre, Tennent Drive, Private Bag 11008, Palmerston North, 4442 New Zealand; Riddet Institute, Massey University, Palmerston North, 4442 New Zealand; Agriculture and Agri-Food Canada, Lethbridge Research Centre, Lethbridge, Alberta T1J 4B1 Canada

**Keywords:** Methanogen, Methane, Ruminant, *Methanosarcina barkeri*

## Abstract

*Methanosarcina* species are the most metabolically versatile of the methanogenic *Archaea* and can obtain energy for growth by producing methane via the hydrogenotrophic, acetoclastic or methylotrophic pathways. *Methanosarcina barkeri* CM1 was isolated from the rumen of a New Zealand Friesian cow grazing a ryegrass/clover pasture, and its genome has been sequenced to provide information on the phylogenetic diversity of rumen methanogens with a view to developing technologies for methane mitigation. The 4.5 Mb chromosome has an average G + C content of 39 %, and encodes 3523 protein-coding genes, but has no plasmid or prophage sequences. The gene content is very similar to that of *M. barkeri* Fusaro which was isolated from freshwater sediment. CM1 has a full complement of genes for all three methanogenesis pathways, but its genome shows many differences from those of other sequenced rumen methanogens. Consequently strategies to mitigate ruminant methane need to include information on the different methanogens that occur in the rumen.

**Electronic supplementary material:**

The online version of this article (doi:10.1186/s40793-015-0038-5) contains supplementary material, which is available to authorized users.

## Introduction

Ruminants are foregut fermenters and have evolved an efficient digestive system in which microbes ferment plant fibre and provide fermentation end-products and other nutrients for growth of the animal [1]. A variety of methanogens can be found in the rumen [2] and ruminant derived methane (CH_4_) accounts for about one quarter of all anthropogenic CH_4_ emissions [3], and is implicated as a driver of global climate change. In terms of their metabolism the rumen methanogens fall into three groups, hydrogenotrophs (*Methanobrevibacter*, *Methanomicrobium* and *Methanobacterium* spp) which convert hydrogen and/or formate to CH_4_, methylotrophs (*Methanosphaera* spp and members of the order *Methanomassiliicoccales*) which produce CH_4_ from methyl compounds such as methanol and methylamines, and acetoclastic methanogens (*Methanosarcina*) which can utilise acetate to produce CH_4_ in addition to the hydrogenotrophic and methylotrophic pathways. Obtaining representative genome sequences from each of the above organisms will be important to understanding the metabolic capacity of these archaea and how they contribute to rumen fermentation processes. Currently, genome sequences are available for five rumen methanogens including strains of *Methanobrevibacter ruminantium* [4], *M. boviskoreani* [5, 6], *Methanobacterium formicicum* [7] and *Thermoplasmatales* archaeon BRNA1 [NCBI Reference Sequence: NC_020892.1]. Development of strategies to reduce CH_4_ emissions from farmed ruminant animals are currently being investigated with methanogen genome sequence information used to inform mitigation strategies based on vaccines and small-molecule inhibitors [8, 9]. Here we present the genome sequence from a rumen acetoclastic methanogen, *Methanosarcina barkeri* CM1.

## Organism information

### Classification and features

**Fig. 1:**
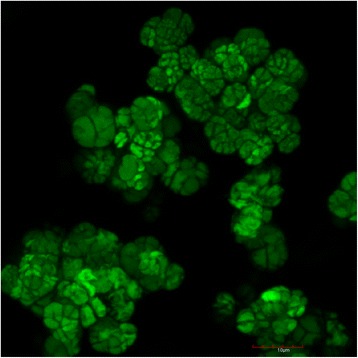
Morphology of *Methanosarcina barkeri* CM1. Micrograph showing aggregates of *Methanosarcina barkeri* CM1 cells captured with sectional depth scanning using an Olympus Fluoview FV1000D Spectral laser confocal scanning inverted microscope, with an UPLSAPO 60X oil objective (1.35 NA). Olympus Fluoview 10-ASW software was used to view fluorescent signals and to generate images. Emission at 635 nm wavelength shows methyl green stain incorporated into nucleic acids within cells and cell clusters. Bar is 10 μm

**Fig. 2:**
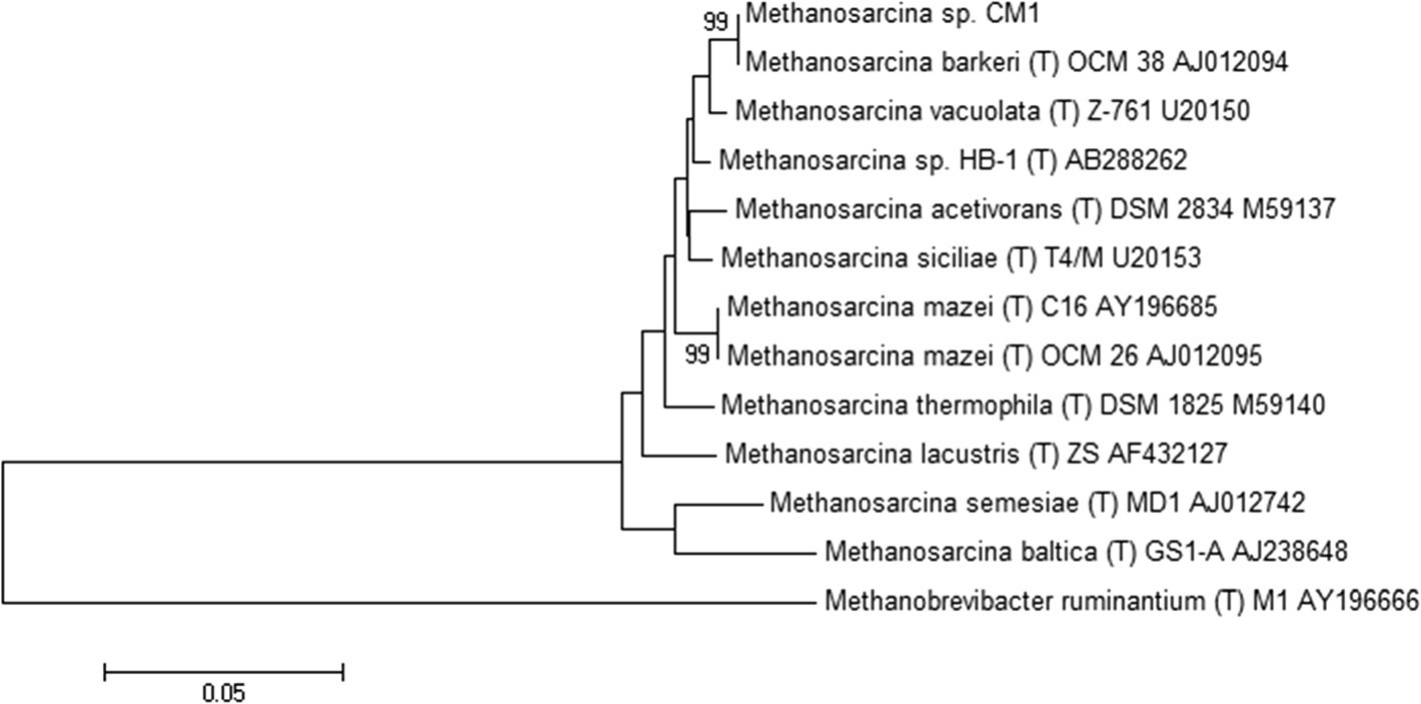
Phylogenetic tree showing the position of CM1 relative to type strains of other *Methanosarcina* species*.* The strains and their corresponding accession numbers are shown. The evolutionary history was inferred using the Neighbor-Joining method [45] with *Methanobrevibacter ruminantium* used as an outgroup. The optimal tree with the sum of branch length = 0.43777587 is shown. The percentage of replicate trees in which the associated taxa clustered together in the bootstrap test (1000 replicates) are shown next to the branches [46]. The tree is drawn to scale, with branch lengths in the same units as those of the evolutionary distances used to infer the phylogenetic tree. The evolutionary distances were computed using the Kimura 2-parameter method [47] and are in the units of the number of base substitutions per site. The rate variation among sites was modeled with a gamma distribution (shape parameter = 1). The analysis involved 13 nucleotide sequences. All positions containing gaps and missing data were eliminated. There were a total of 1081 positions in the final dataset. Evolutionary analyses were conducted in MEGA5 [48]

*Methanosarcina* sp. CM1 was isolated from the rumen of a New Zealand Friesian cow grazing a ryegrass/clover pasture [10]. CM1 grew as large cell aggregates in broth culture and showed the characteristic morphology associated with *Methanosarcina barkeri* [11] (Fig. 1). It was described as non-motile, and able to grow and produce methane from H_2_/CO_2_, acetate, methanol and methylamines. Growth occurred between 30° and 45 °C, and at pH 5.0 to 7.4. Rumen fluid was required for growth. The 16S rRNA gene from CM1 is 99 % similar to that of the *Methanosarcina barkeri* type strain MS (DSM 800) (Fig. 2) which was isolated from a sewage sludge digester [12, 13], and as such CM1 can be considered as a strain of *M. barkeri*. *M. barkeri* is found at high densities in anaerobic digesters and anoxic marine and freshwater sediments, but there have been several reports describing *Methanosarcina* from the rumen although these organisms were not characterized [14, 15]. In addition, non-rumen strains of *M. barkeri* have been used in co-culture studies with rumen anaerobic fungi [16] and ciliate protozoa [17, 18]. Characteristics of *Methanosarcina barkeri* CM1 are shown in Table 1 and Additional file 1.

**Table 1:** Classification and general features of *Methanosarcina barkeri* CM1

MIGS ID	Property	Term	Evidence code^a^
	Classification	Domain: Archaea	TAS [49]
		Phylum: *Euryarchaeota*	TAS [50]
		Class: *Methanococci*	TAS [51, 52]
		Order: *Methanosarcinales*	TAS [51, 53]
		Family: *Methanosarcinaceae*	TAS [24, 54]
		Genus: *Methanosarcina*	TAS [55, 56]
		Species: *Methanosarcina barkeri*	TAS [10]
		strain: CM1	
	Gram stain	Positive	TAS [12]
	Cell shape	Irregular	TAS [10]
	Motility	Non-motile	TAS [10]
	Sporulation	Not reported	IDA
	Temperature range	30-45 °C	TAS [10]
	Optimum temperature	40 °C	TAS [10]
	pH range; Optimum	5.0-7.4; 6.8	TAS [10]
	Carbon source	CO_2_, Acetate	IDA
MIGS-6	Habitat	Bovine rumen	TAS [10]
MIGS-6.3	Salinity	Not reported	
MIGS-22	Oxygen requirement	Anaerobic	IDA
MIGS-15	Biotic relationship	Symbiont	TAS [10]
MIGS-14	Pathogenicity	Non-pathogen	NAS
MIGS-4	Geographic location	Palmerston North, New Zealand	IDA
MIGS-5	Sample collection	Not reported	
MIGS-4.1	Latitude	-40.35 (40°21'00"S)	IDA
MIGS-4.2	Longitude	+175.61 (175°36'36"E)	IDA
MIGS-4.4	Altitude	30 M	IDA

^a^Evidence codes - IDA: Inferred from Direct Assay; TAS: Traceable Author Statement (i.e., a direct report exists in the literature); NAS: Non-traceable Author Statement (i.e., not directly observed for the living, isolated sample, but based on a generally accepted property for the species, or anecdotal evidence). These evidence codes are from the Gene Ontology project [57]

## Genome sequencing information

### Genome project history

*Methanosarcina barkeri* CM1 was selected for genome sequencing on the basis of its phylogenetic position relative to other methanogens isolated from the rumen. A summary of the genome project information is shown in Table 2.

**Table 2:** Project information

MIGS ID	Property	Term
MIGS-31	Finishing quality	High-quality, closed genome
MIGS-28	Libraries used	454 3 kb mate paired-end library, Illumina paired-end 170 bp insert library
MIGS-29	Sequencing platforms	454 GS FLX Titanium chemistry, Illumina
MIGS-31.2	Fold coverage	97× (454), 224× (Illumina)
MIGS-30	Assemblers	Newbler, Spades
MIGS-32	Gene calling method	Glimmer and BLASTX
	Locus tag	MCM1
	Genbank ID	CP008746
	Genbank date of release	June 3, 2015
	GOLD ID	Gp0007672
MIGS 13	Source material identifier	CM1
	Project relevance	Ruminant methane emissions

### Growth conditions and genomic DNA preparation

*Methanosarcina barkeri* CM1 was grown in BY medium [19] with added SL10 Trace Elements solution (1 ml added l^−1^ [20], 20 mM sodium acetate, 60 mM sodium formate and Vitamin 10 solution (0.1 ml added to 10 ml culture before inoculation) [4]. H_2_ was supplied as the energy source by pumping the culture vessels to 180 kPa over pressure with an 80:20 mixture of H_2_:CO_2_. Genomic DNA was extracted from freshly grown cells using a modified version of a liquid N_2_ and grinding method as described previously [6].

### Genome sequencing and assembly

The complete genome sequence of CM1 was determined using pyrosequencing of 3Kb mate paired-end sequence libraries using a 454 GS FLX platform with Titanium chemistry (Macrogen, Korea). Pyrosequencing reads provided 97× coverage of the genome and were assembled using the Newbler assembler version 2.7 (Roche 454 Life Sciences, USA). The Newbler assembly resulted in 85 contigs across 9 scaffolds. Gap closure was managed using the Staden package [21] and gaps were closed using additional Sanger sequencing by standard and inverse PCR based techniques. In addition, CM1 genomic DNA was sequenced using the Illumina HiSeq 2000 platform (Beijing Genomics Institute, China) which provided 223× genome coverage. Illumina reads were assembled using the Spades assembler version 3.0 [22] and combined with the Newbler assembly using the Staden package. Assembly validation was confirmed by pulsed-field gel electrophoresis as described previously [6].

### Genome annotation

The procedure for genome annotation was as described previously for *Methanobrevibacter* sp. [4, 6], and the CM1 genome sequence was prepared for NCBI submission using Sequin. The adenine residue of the start codon of the Cdc6-1 replication initiation protein A (MCM1_0001) gene was chosen as the first base for the CM1 genome. The nucleotide sequence of the *Methanosarcina barkeri* CM1 chromosome has been deposited in Genbank under accession number CP008746.

## Genome properties

The genome of *Methanosarcina barkeri* CM1 consists of a single 4,501,171 basepair (bp) circular chromosome with an average G + C content of 39 %. A total of 3656 genes were predicted, 3523 of which were protein-coding genes, representing 70 % of the total genome sequence. A COG category was assigned to 2267 of the protein-coding genes. The properties and statistics of the genome are summarized in Tables 3 and 4. As with the other sequenced *Methanosarcina* strains CM1 has dual origins of replication (MCM1_001 and MCM1_3593, 95 kb apart) surrounded by conserved genes [23]. The CM1 genome has neither plasmid nor prophage sequences, but does contain three clusters of CRISPR genes associated with CRISPR repeat regions, and three type I restriction/modification systems.

**Table 3:** Genome statistics

Attribute	Value	% of Total
Genome size (bp)	4,501,171	100.00
DNA coding (bp)	3,149,919	69.98
DNA G + C (bp)	1,763,740	39.18
DNA scaffolds	1	
Total genes	3,655	100.00
Protein coding genes	3,523	96.39
RNA genes	69	1.89
Pseudo genes	63	1.72
Genes with function prediction	2,410	65.94
Genes assigned to COGs	2,267	64.35
Genes with Pfam domains	2,953	80.79
Genes with signal peptides	358	10.16
Genes with transmembrane helices	881	25.01
CRISPR repeats	3

**Table 4:** Number of genes associated with the 25 general COG functional categories

Code	Value	% of total^a^	Description
J	158	4.48	Translation
A	1	0.03	RNA processing and modification
K	112	3.18	Transcription
L	126	3.58	Replication, recombination and repair
B	2	0.06	Chromatin structure and dynamics
D	15	0.43	Cell cycle control, mitosis and meiosis
Y	-	-	Nuclear structure
V	76	2.16	Defense mechanisms
T	63	1.79	Signal transduction mechanisms
M	99	2.81	Cell wall/membrane biogenesis
N	16	0.45	Cell motility
Z	-	-	Cytoskeleton
W	-	-	Extracellular structures
U	18	0.51	Intracellular trafficking and secretion
O	96	2.72	Posttranslational modification, protein turnover, chaperones
C	223	6.33	Energy production and conversion
G	81	2.30	Carbohydrate transport and metabolism
E	221	6.27	Amino acid transport and metabolism
F	54	1.53	Nucleotide transport and metabolism
H	109	3.09	Coenzyme transport and metabolism
I	30	0.85	Lipid transport and metabolism
P	138	3.92	Inorganic ion transport and metabolism
Q	48	1.36	Secondary metabolites biosynthesis, transport and catabolism
R	368	10.44	General function prediction only
S	213	6.04	Function unknown
-	1256	35.67	Not in COGs

^a^The total is based on the total number of protein coding genes in the annotated genome

## Insights from the genome sequence

The genome of *Methanosarcina barkeri* CM1 is compared with genomes of other sequenced methanogens from the genus *Methanosarcina* in Table 5. Overall, the gene content of the CM1 genome is very similar to that of *Methanosarcina barkeri* Fusaro, but gene organization shows very little synteny.

**Table 5:** Genomes of *Methanosarcina* species from various anaerobic environments

Species	Isolation source	Genome size (Mb)	Accession #	CDS	% GC	Reference
*Methanosarcina barkeri* CM1	Bovine rumen	4.50	CP008746	3,524	39.2	This report
*Methanosarcina barkeri* Fusaro	Freshwater sediment	4.87	NC_007355	3,758	39.2	[23]
*Methanosarcina acetivorans* C2A	Marine sediment	5.75	AE010299	4,721	42.7	[32]
*Methanosarcina mazei* Go1	Sewage	4.10	AE008384	3,398	41.5	[58]
*Methanosarcina mazei* Tuc01	Freshwater sediment	3.42	CP004144	3,395	42.5	[59]

### Methanogenesis

**Fig. 3:**
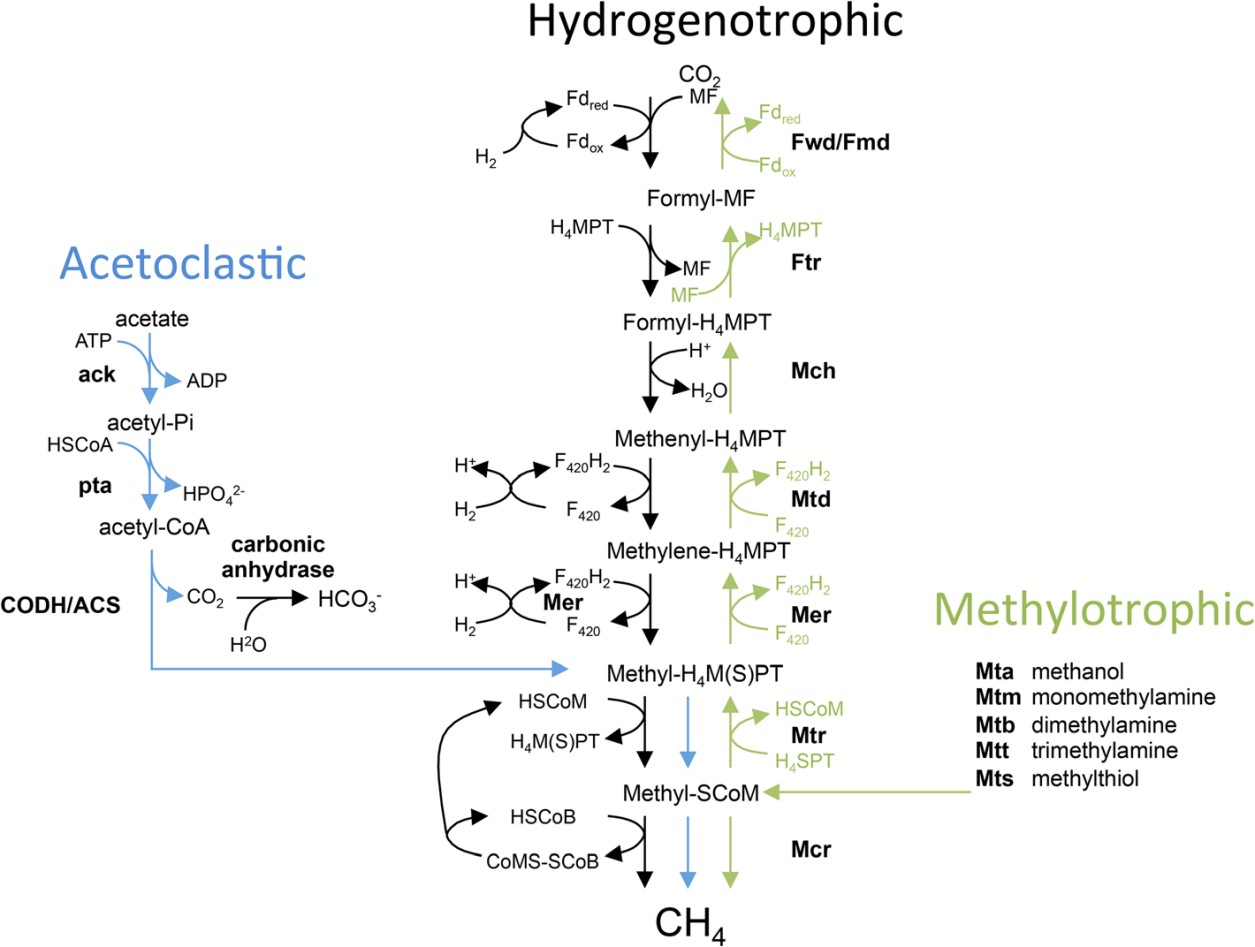
The three methanogenesis pathways inferred from the genome of *Methanosarcina barkeri* CM1

*Methanosarcina* species are the most metabolically versatile of the methanogenic archaea [24] and can obtain energy for growth by producing methane via three different pathways (Fig. 3). Methane can be derived from the reduction of CO_2_ with hydrogen (hydrogenotrophic pathway), from the methyl group of acetate (acetoclastic pathway), or from the methyl group of methanol, methylamines or methylthiols (methylotrophic pathway). Each pathway culminates in the transfer of a methyl group to coenzyme M and the subsequent reduction to methane. The bioenergetics of aceticlastic methanogens have been recently reviewed [25, 26], and a metabolic reconstruction presented for *M. barkeri* Fusaro [27]. There is evidence that the genes essential to both the acetoclastic and methyoltrophic pathways were horizontally acquired during evolution of the *Methanosarcinaceae* [28–30]. Although acetoclastic methanogenesis contributes approximately two-thirds of the methane in the biosphere [31], acetate is not metabolized to methane to any significant extent in the rumen [2]. CM1 has a full complement of genes for all three methanogenesis pathways and as with other *Methanosarcina* species several genes are present as multiple copies [32]. Unlike many hydrogenotrophic methanogens, CM1 does not have the gene for [Fe]-hydrogenase dehydrogenase (*hmd*), or the genes that encode methyl coenzyme M reductase II (*mrt*), however it does have genes for formate dehydrogenase (MCM1_3047-3048) although CM1 and other *M. barkeri* strains are unable to use formate [10, 23]. The pathway for coenzyme M biosynthesis differs from that found in other sequenced rumen methanogens which belong to the order *Methanobacteriales* [33]. Consequently methanogen inhibitors targeting coenzyme M biosynthesis would not be expected to work against all rumen methanogen species. *M. barkeri* is the organism in which the 22nd amino acid (pyrrolysine) was discovered during examination of the methyltransferases required for methane formation from methylamines [34]. Biosynthesis of this amino acid requires specialized enzymes together with a specific aminoacyl-tRNA synthetase [35], and the genes encoding these (*pyl*SBCD) are found in CM1 (MCM1_2535-2538).

### Cell envelope

The majority of rumen methanogens belong to the family *Methanobacteriaceae* and have a characteristic pseudomurein-containing cell wall [4]. The cell surface of *Methanosarcina* sp. is different and electron microscopy shows a protein S-layer surrounding the cytoplasmic membrane. A major S-layer protein has been identified in three *Methanosarcina* species and used to define a family of proteins [36, 37]. All paralogs possess signal peptides and one or two DUF1608 (Pfam accession PF07752) domains. Both *M. barkeri* Fusaro and CM1 possess nine proteins containing this domain. Gene expression studies show that a single DUF1608 domain-containing protein is abundantly expressed in both *M. acetivorans* (MA0829) and *M. barkeri* (Mbar_A1758), and is among the most highly expressed of all proteins in the cell [37]. A similar protein is predicted from the CM1 genome (MCM1_2018, 84 % amino acid identity to the product of Mbar_A1758), and likely to be the major S-layer protein for this strain. The DUF1608 domain from MA0829 has been crystallised and was used to propose an elegant model of the *Methanosarcina* S-layer structure [38]. *Methanosarcina* cells can form large aggregates (Fig. 1) mediated by the production of methanochondroitin, a polymer composed of uronic acid and N-acetylgalactosamine residues [39]. While the steps in methanochondroitin biosynthesis have been determined, the genes involved have yet to be identified. These aggregates are observed to separate into single cells and CM1 encodes eleven proteins with disaggregatase-related domains (Pfam accession PF08480). The CM1 genome has four oligosaccharyl transferase genes, three of which (MCM1_1841-1843) are at the end of a large gene cluster that contains 14 glycosyl transferases and is likely to be involved in polysaccharide biosynthesis (MCM1_1841-1889). The fourth oligosaccharyl transferase is associated with a smaller gene cluster containing glycosyl transferases, methyltransferases and transporters (MCM1_2113-2123). A third cluster of polysaccharide biosynthesis genes is found at MCM1_2831-2857. CM1 also encodes a secreted protein (MCM_2974) containing a glycoside hydrolase family 18 (chitinase) domain that is not found in *M. barkeri* Fusaro which may be involved in mediating interaction with rumen anaerobic fungi. Like many other archaea, CM1 has an identifiable archaella (archaeal flagella) operon (FlaB-FlaJ, MCM1_1947-1953), together with a cluster of chemotaxis genes (MCM1_3655-3662) [40, 41]. However, motility has never been observed in any *Methanosarcina* species and thus the function of these genes remains unknown.

*Methanosarcina barkeri* has been reported to fix nitrogen [42] and sets of nitrogenase genes are found in *Methanosarcina* genomes. CM1 contains two different *nif* operons comprising nitrogenase and nitrogenase cofactor biosynthesis genes that match to those reported from *M. barkeri* strain 227 [43]. These are a molybdenum- and iron-containing nitrogenase (MCM1_2924-2930) and a vanadium- and iron-containing nitrogenase (MCM1_1063-1072). However, it does not have the genes for the third type, the iron-only nitrogenase that is found in *M. acetivorans* and *M. barkeri* Fusaro. Electron micrographs of *M. barkeri* log phase cells [11] show the presence of numerous electron-dense granules in the cytoplasm. In *M. thermophila* similar granules were found to contain glycogen [44], and CM1 has several genes predicted to encode the enzymes necessary for the biosynthesis and degradation of this reserve polysaccharide. CM1 does not have the genes for gas vesicle biosynthesis that are found in the *M. barkeri* Fusaro genome [23], but it does have genes for the two-subunit acetyl-CoA synthetase (MCM1_1658 and 2708) that have been lost from the Fusaro strain.

## Conclusion

The genome of *Methanosarcina barkeri* CM1 is very similar to that of the freshwater sediment isolate *M. barkeri* Fusaro, but markedly different from the dominant rumen methanogens, most of which are members of the family *Methanobacteriaceae*. CM1 has a much larger genome and its sequence provides new insights into the metabolic versatility of rumen methanogens. With its ability to use three different methanogenesis pathways, *M. barkeri* appears to be a generalist able to occupy a range of different environments but is not particularly at home in the rumen. This is in contrast to the more specialised rumen methanogens, such as the *Methanobrevibacter* species which dominate the rumen environment. Analysis of the methanogenesis pathway and the cell envelope have been important for the design of methane mitigation strategies targeting rumen methanogens, but differences highlighted from the CM1 genome stress the need to include information from all rumen methanogens in the design of mitigation approaches.

## Additional file

Additional file 1: Table S1. Associated MIGS record for CM1, which links to the SIGS supplementary content website.
