# Supplementary material for: The complete genome sequence of the rumen methanogen Methanosarcina barkeri CM1
Source: Stand Genomic Sci. 2015 Aug 19;10:57. doi: 10.1186/s40793-015-0038-5 (PMC4582637; doi:10.1186/s40793-015-0038-5)
Supplement: Additional file 1: Table S1. — Associated MIGS record for CM1, which links to the SIGS supplementary content website. [file 40793_2015_38_MOESM1_ESM.pdf]

Appendix 1

Table S1. Associated MIGS record

MIGS-ID	field name	description
**MIGS-1**	Submit to INSDC/Trace archives	
**1.1**	PID	
**1.2**	Trace Archive	
**MIGS-2**	MIGS CHECK LIST TYPE	
**MIGS-3**	Project Name	*Methanosarcina barkeri* CM1
**MIGS-4**	Geographic Location	Palmerston North, New Zealand
**4.1**	Latitude	-40.35 (40°21'00"S)
**4.2**	Longitude	+175.61 (175°36'36"E)
**4.3**	Depth	NA
**4.4**	Altitude	30m
**MIGS-5**	Time of Sample collection	Not reported
**MIGS-6**	Habitat (EnvO)	Cow rumen
**6.1**	temperature	37-39°C
**6.2**	pH	6.8
**6.3**	salinity	
**6.4**	chlorophyll	
**6.5**	conductivity	
**6.6**	light intensity	
**6.7**	dissolved organic carbon (DOC)	
**6.8**	current	
**6.9**	atmospheric data	
**6.10**	density	
**6.11**	alkalinity	
**6.12**	dissolved oxygen	

**6.13**	particulate organic carbon (POC)	
**6.14**	phosphate	
**6.15**	nitrate	
**6.16**	sulfates	
**6.17**	sulfides	
**6.18**	primary production	
**MIGS-7**	Subspecific genetic lineage	Strain CM1
**MIGS-9**	Number of replicons	1
**MIGS-10**	Extrachromosomal elements	0
**MIGS-11**	Estimated Size	4501171bp
**MIGS-12**	Reference for biomaterial or Genome report	
**MIGS-13**	Source material identifiers	Bovine rumen contents
**MIGS-14**	Known Pathogenicity	Not known as a pathogen
**MIGS-15**	Biotic Relationship	Rumen symbiont
**MIGS-16**	Specific Host	Bovine
**MIGS-17**	Host specificity or range (taxid)	Ruminants
**MIGS-18**	Health status of Host	Healthy
**MIGS-19**	Trophic Level	Methanogen
**MIGS-22**	Relationship to Oxygen	Strict anaerobe
**MIGS-23**	Isolation and Growth conditions	Cultured from a cow rumen sample
**MIGS-27**	Nucleic acid preparation	Freeze grinding
**MIGS-28**	Library construction	3Kb mate paired end (454)/ 170bp insert paired-end (illumina)
**28.1**	Library size	436687130bp (454)/ 1007000100bp (illumina)
**28.2**	Number of reads	1494961 (454)/ 11188890 (illumina)
**28.3**	vector	NA

**MIGS-29**	Sequencing method	Pyrosequencing/illumina
**MIGS-30**	Assembly	
**30.1**	Assembly method	Newbler, Spades
**30.2**	estimated error rate	
**30.3**	method of calculation	
**MIGS-31**	Finishing strategy	
**31.1**	Status	Closed
**31.2**	coverage	97x (454)/224x (illumina)
**31.3**	contigs	1
**MIGS-32**	Relevant SOPs	
**MIGS-33**	Relevant e-resources	

Appendix 2.

Table S2. GenBank Accession Summary

GenBank Accession	Summary
CP008746	CP008746.1 is a bacterial sequences record containing circular, double-stranded DNA (4,501,171 bases) from Methanosarcina barkeri strain CM1. The record was created on June 3, 2015. It contains 7,248 features, including 3655 genes with 632 distinct annotations, 3523 DNA coding regions, 9 rRNA features (16S ribosomal RNA, 5S ribosomal RNA and 23S ribosomal RNA) and 60 tRNA features coding for 20 distinct amino acids.
AE010299	AE010299.1 is a bacterial sequences record containing circular, double-stranded DNA (5,751,492 bases) from Methanosarcina acetivorans strain C2A. The record was created on April 3, 2002 and last updated October 1, 2014. It contains 9,213 features, including 4662 genes with 687 distinct annotations, 4540 DNA coding regions, 10 rRNA features (16S ribosomal RNA, 5S ribosomal RNA, 7S ribosomal RNA and 23S ribosomal RNA).
CP004144	CP004144.1 is a bacterial sequences record containing circular, double-stranded DNA (3,427,949 bases) from Methanosarcina mazei strain Tuc01. The record was created on February 22, 2013 and last updated January 31, 2014. It contains 6,746 features, including, 3252 DNA coding regions, 9 rRNA features (16S ribosomal RNA, 5S ribosomal RNA and 23S ribosomal RNA) and 40 tRNA features coding for 19 distinct amino acids.
AE008384	AE008384.1 is a bacterial sequences record containing circular, double-stranded DNA (4,096,345 bases) from Methanosarcina mazei strain Go1. The record was created on May 20, 2002 and last updated January 30, 2014. It contains 6,877 features, including 3438 genes with 145 distinct annotations, 3371 DNA coding regions, 10 rRNA features (LSU Ribosomal RNA, SSU ribosomal RNA and 5S Ribosomal RNA) and 57 tRNA features coding for 20 distinct amino acids.
NC_020892	NC_020892.1 is a contig record containing circular, double-stranded DNA (1,461,105 bases) from Thermoplasmatales archaeon strain BRNA1. The record was created on April 3, 2013

	and last updated December 18, 2014. It contains 1 feature.
NC_007355	NC_007355.1 is a contig record containing circular, double-stranded DNA (4,837,408 bases) from Methanosarcina barkeri str. strain Fusaro. The record was created on August 22, 2005 and last updated December 16, 2014. It contains 1 feature.
